# Over expression of CDK4 and MDM2 in a patient with recurrent ALK-negative mediastinal inflammatory myofibroblastic tumor: A case report

**DOI:** 10.1097/MD.0000000000019577

**Published:** 2020-03-20

**Authors:** Tien-Chi Hou, Pao-Shu Wu, Wen-Yu Huang, Yi-Ting Yang, Kien Thiam Tan, Shih-Hua Liu, Yu-Jen Chen, Shu-Jen Chen, Ying-Wen Su

**Affiliations:** a Department of Radiation Oncology; b Department of Pathology; c Laboratory of Good Clinical Research Center, Mackay Memorial Hospital, Tamsui Branch, New Taipei City; d ACT Genomics Co., Ltd.; e Division of Hematology and Medical Oncology, Department of Internal Medicine, Mackay Memorial Hospital, Taipei, Taiwan.

**Keywords:** cyclin dependent Kinase 4, inflammatory myofibroblastic tumor, inflammatory pseudotumor, malignant transformation, mouse double minute 2 homolog, next-generation sequencing

## Abstract

**Rationale:**

The diagnosis of anaplastic lymphoma kinase (*ALK*)-negative inflammatory myofibroblastic tumors (IMT) remains challenging because of their morphological resemblance with spindle cell sarcoma with myofibroblastic characteristics.

**Patient concerns:**

A 69-year-old female patient presented with loco-regional recurrent IMT several times within 8 years after primary treatment and neck lymph node metastasis 3.5 years after last recurrence.

**Diagnosis:**

The primary, recurrence, and lymph node metastasis lesions were diagnosed as ALK-negative IMTs based on the histopathological features.

**Interventions:**

Biopsy samples were obtained during repeated surgeries and evaluated for genomic alterations during first and recurrent presentations. The evaluation was done using pathway-driven massive parallel sequencing, and genomic alterations between primary and recurrent tumors were compared.

**Outcomes:**

Copy number gains and overexpression of mouse double minute 2 homolog (*MDM2*) and cyclin dependent kinase 4 (*CDK4*) were observed in the primary lesion, and additional gene amplification of Discoidin Domain Receptor Tyrosine Kinase 2 (*DDR2*), Succinate Dehydrogenase Complex II subunit C (*SDHC*), and thyroid stimulating hormone receptor (*TSHR*) Q720H were found in the recurrent tumors. Metastases to the neck lymph node were observed 3.5 years after recurrence.

**Lessons:**

Our results indicated genetic evolution in a microscopically benign condition and highlighted the importance of molecular characterization of fibro-inflammatory lesions of uncertain malignant potential.

## 1 Introduction

Inflammatory myofibroblastic tumors (IMTs) are traditionally considered as idiopathic benign inflammatory lesions. They are characterized by spindle cell proliferation in a spiral pattern, and infiltration by inflammatory cells including plasma cells, lymphocytes, and macrophages.^[1]^ Due to their histological heterogeneity, IMTs have also been described as inflammatory pseudotumors or plasma cell granulomas. Surgical resection is the primary treatment for IMTs; however, unlike other benign neoplasms, invasion to surrounding organs, recurrence, and metastasis to lymph nodes and distant organs has been often reported in cases that underwent complete resection.^[2–5]^ Therefore, in 2006, World Health Organization (WHO) defined IMTs as intermediary lesions with the potential of turning recurrent and malignant.^[6]^

Given the unpredictable and aggressive clinical behavior of IMTs, it is challenging for clinicians to develop an effective treatment strategy. Clinically, tumors >3 cm with focal and vascular invasion and nuclear pleomorphism are suggestive of malignant behavior and poor prognosis.^[2]^ However, whether the inflammatory microenvironment of the tumor or the malignant transformation is responsible for recurrence in IMTs remains controversial.

We hypothesized that genetic changes determined the malignancy of the lesion and could serve as a reference to elucidate their prognosis. In this report, we explored the genetic evolution of a recurrent IMT through a pathway-driven massive parallel genetic sequencing approach.

## 2 Case report

**Figure 1:**
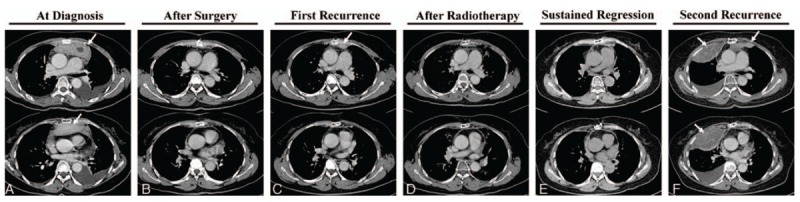
Computed tomography scans of the thorax of the patient. A: at diagnosis; B: after the first surgery; C: recurrence at 9 months after surgery; D: regression after radiotherapy; E: sustained regression for 7.5 years after surgery; F: second recurrence at 8 years after surgery. White arrows indicate tumors.

In 2007, a 57-year-old female patient presented with a gradual onset of tightening sensation around the retrosternal region, without fever or weight loss. Initial investigations by computed tomography (CT) scan demonstrated a bulky retrosternal mass (9.5 × 6 × 3 cm) at the anterior mediastinum with left pleural invasion, and left pleural and pericardial effusions (Fig. 1A). She underwent surgical removal of the tumor, and a biopsy was taken for further evaluation.

**Figure 2:**
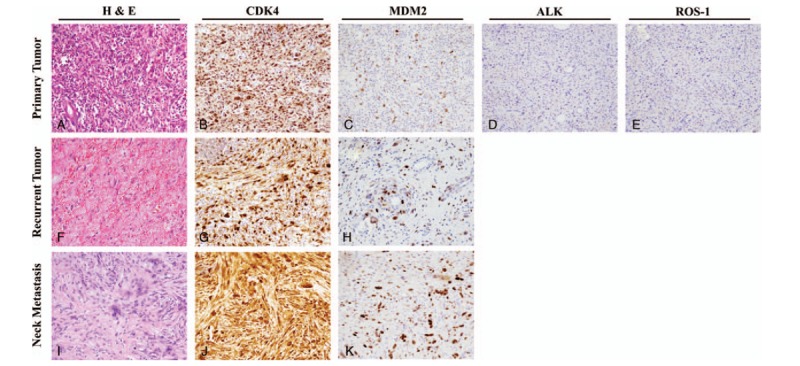
Histology of inflammatory pseudotumor from the primary, recurrent, and neck metastatic sites. The primary tumor was composed of spindle cells with heavily infiltrated inflammatory cells including lymphocytes, eosinophils, and plasma cells (A, H&E,); the tumor cells did not stain positive for anaplastic lymphoma kinase (ALK) (D) and ROS-1 (E); tumor cells showed nuclear overexpression of CDK4 (B) and MDM2 (C). The recurrent tumor showed lesser inflammatory infiltrate but presented with higher cellular pleomorphism and atypical cytological features (F). Strong expression of CDK4 and MDM2 in the recurrent tumor can be observed in panel G and H, respectively. Although the tumor samples from the neck lymph node metastasis showed a more pleomorphic pattern (I), CDK4 and MDM2 were consistently overexpressed (J and K, respectively). All images were taken at 200× magnification.

Upon microscopic examination, the mass was found to be composed of proliferating spindle cells and presented with heavy infiltration of inflammatory cells, including plasma cells, neutrophils, lymphocytes, and histiocytes. Parts of the tumor were edematous, hemorrhagic, and manifested cystic changes. Immunohistochemically, some of the spindle cells were positive for α-smooth muscle actin (ASMA), but all were negative for anaplastic lymphoma kinase (ALK), CD30, CD15, and S100. Based on these findings, the tumor mass was diagnosed as IMT (Fig. 2A–E).

Nine months after the surgery, the tumor recurred at the left anterior mediastinum. This time the tumor was treated with radiotherapy (50.4 Gy toward the surgical bed with 10.8 Gy boost to the recurrent tumor) and regressed after the treatment. The patient remained recurrence-free until 8 years after the surgery. In 2015 (at the age of 65), the tumor recurred as a rapidly growing retrosternal mass (14 × 13.6 × 4.9 cm) around the left anterior mediastinum and right anterior pleural space. The patient underwent a second surgery, and the biopsy report showed similar microscopic findings as in the previous biopsy, but with increased atypical cytology, hemorrhage, and necrosis. Reduced inflammatory infiltration and rich myxoid pattern were also observed (Fig. 2F). The patient was healthy until 3.5 years after the second surgery when metastases to the neck lymph nodes were noted (Fig. 2I).

Through this work, we aimed to study the genetic alteration during tumor progression by using a commercially available multiplexed gene sequencing panel (ACTOnco, ACT Genomics Co., Ltd. Taipei, Taiwan). The kit was used to detect mutations in 409 genes related to cancer diagnosis and prognosis.

The study was approved by the Ethics Committee of Mackay Memorial Hospital (Approval No. 16MMHIS070e). Written informed consent was obtained before collecting the samples in compliance with the Ethics Committee's recommendations.

Immunohistochemistry was performed using the BenchMark ULTRA slide staining system (Ventana Medical Systems: Arizona, AZ, USA). Staining was performed as per the standard protocol using following primary antibodies: anti-ALK antibody (Zymed, San Francisco, CA; 1:100 dilution), anti-MDM2 antibody (Santa Cruz Biotechnology, Santa Cruz, CA; 1:50 dilution), and anti-CDK4 (Santa Cruz Biotechnology, Santa Cruz, CA; 1:50 dilution). To evaluate the cell proliferation rate, anti-Ki-67 antibody (MIB-1, Dako; 1:20 dilution) was used.

Genomic DNA (gDNA) was extracted from formalin-fixed, paraffin-embedded tumor tissues using the QIAamp DNA FFPE Tissue Kit (Qiagen Inc., Valencia, CA). Blood samples were collected in Ethylenediaminetetraacetic acid-coated tubes and genomic DNA was isolated using the Gentra Puregene Blood Kit (Qiagen Inc. Valencia, CA) according to the manufacturer's protocol. DNA was quantified by the Quant-iT dsDNA HS Assay (Invitrogen, CA) and tested for integrity using the Fragment Analyzer (Advanced Analytical Technologies, Iowa, IA) according to the manufacturer's protocol.

Extracted DNA from each sample was amplified by multiplex polymerase chain reaction using 4 pools of 15,992 primer pairs (Ion AmpliSeq Comprehensive Cancer Panel, Thermo Fisher Scientific, Massachusetts, MA) targeting all coding exons of 409 cancer-related genes (1688650 target bases). Amplicons were ligated with barcoded libraries, conjugated with sequencing beads by emulsion polymerase chain reaction, and enriched using IonChef (Life Technologies, Carlsbad, CA). Sequencing was performed on the Ion Proton Sequencer with the Ion PI chip (Life Technologies).

Raw data generated by the sequencer were mapped to the hg19 reference genome using Ion Torrent Suite (v. 5.10). Coverage depth was calculated using the Torrent Coverage Analysis plug-in. Single nucleotide variants (SNVs) and short insertion/deletions (INDELs) were identified using the Torrent Variant Caller plug-in (version 5.10). For variant annotation, variant effect predictor (VEP) and databases, including COSMIC: v.86 and 1000 Genomes: phase3, were used. Variants with coverage <25 or mutant allele frequency <5% were filtered out.

Copy number variations were predicted using the following steps. First, amplicons with read counts in the lowest 5th percentile, and those with a coefficient of variation ≥0.3 were removed. Each pool was normalized to correct the samples generated from different amplicon pool designs. Thereafter, ONCOCNV^[7]^ was applied to normalize total amplicon number, GC content of each amplicon region, amplicon length, technology-related biases, and to segment the sample with a gene-aware model. These steps were applied to establish the copy number baseline from peripheral blood mononuclear cells (PBMCs) isolated from blood as well as to analyze the copy number variations from biopsy samples of different stages of IMT.

**Figure 3:**
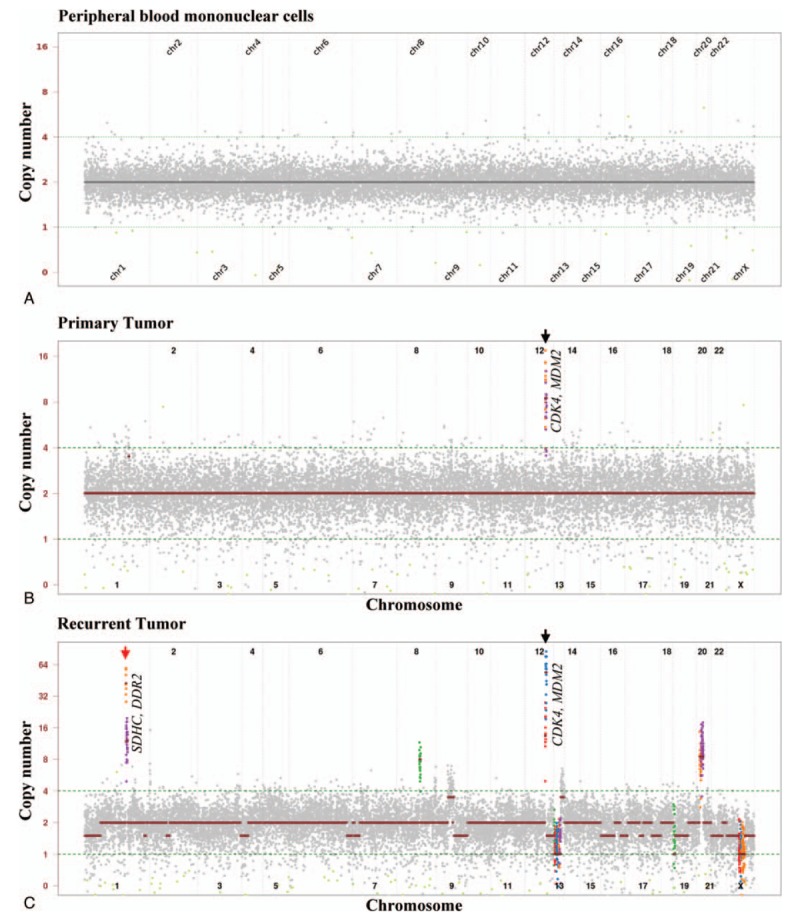
Copy number variation analysis in peripheral blood mononuclear cells (PBMCs) and primary and recurrent inflammatory myofibroblastic tumors (IMTs). Graphical view of amplified regions of chromosome 1 and 12 are highlighted by red circles. The genes encoded in the amplified regions are indicated. *CDK4* = Cyclin Dependent Kinase 4, *DDR2* = Discoidin Domain Receptor Tyrosine Kinase 2, *MDM2* = Mouse Double Minute 2 homolog, *SDHC* = Succinate Dehydrogenase Complex II subunit C.

Massively parallel sequencing for a panel of 409 cancer-related genes identified 6 germline mutations (*PARP1* p.V69I, *ATR* p.S1007N, *GRM8* p.T97A, *MLLT10* p.G409R, *TCF7L2* p.N185S, *SMARCA4* p.A321T) and 1 somatic mutation (*TSHR* p.Q720H) in the recurrent IMT samples (Table 1). Copy number analysis revealed copy number gains for cyclin dependent kinase 4 (*CDK4*) and mouse double minute 2 homolog (*MDM2*) in the primary IMT, and of *CDK4*, *MDM2*, Discoidin Domain Receptor Tyrosine Kinase 2 (*DDR2*), and Succinate Dehydrogenase Complex II subunit C (*SDHC*) in the recurrent IMT (Fig. 3). The findings were supported by CDK4 and MDM2 overexpression results from the immunochemical studies of primary and recurrent IMT lesions (Fig. 2).

**Table 1:**
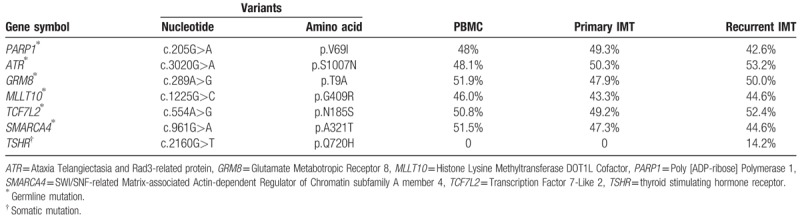
Single nucleotide variants and short insertion/deletions in peripheral blood mononuclear cells (PBMCs) and primary and recurrent inflammatory myofibroblastic tumor (IMTs) in a comprehensive cancer panel sequencing screen containing 409 cancer-related exons (percentages indicate frequency of the mutation).

## 3 Discussion

In this study, we hypothesized that genetic alteration may drive the clinical aggressive behavior of IMTs. Using a comparative pathway-driven massively parallel sequencing approach, we were able to identify gains in copy numbers of *CDK4* and *MDM2* in the primary IMT. We presume that this increase in copy number might contribute to the rapid, aggressive clinical presentation within 9 months of the first surgery. In the recurrent lesion that occurred after 8 years of the radiotherapy, copy number amplification was observed for *CDK4*, *MDM2*, *DDR2*, and *SDHC* gene loci, and 1 new mutation was identified at *TSHR* p.Q720H. These results clearly show that in our case, IMT underwent genetic evolution over time, with a concurrent recurrence, and malignant transformation.

To date, limited scientific literature is available for a comprehensive understanding of the genetic characteristics of IMTs. Cytogenetic studies have shown that the rearrangement and overexpression of the *ALK* gene are the most frequent molecular abnormalities in 34% to 70% of IMTs.^[8–13]^ Other molecular derangements reported in IMTs include gene fusion involving *ROS1*, *NTRK3*, *RET*, and *PDGFRβ*.^[8,12,13]^*ALK*-positive IMTs are often correlated with an increased probability of recurrence; however, in our study, the primary and recurrent tumors were immunohistochemically negative for *ALK* expression.

The diagnosis of *ALK*-negative IMTs is challenging as their morphology is strikingly similar to the spindle cell sarcoma with myofibroblastic characteristics, including their inflammatory features. In our case, the primary IMT exhibited copy number gain and positive immunoreactivity for *MDM2* and *CDK4*, the well-established biomarkers for well-differentiated and dedifferentiated liposarcomas (WDLS and DDLS).^[14]^ A previous study, using immunohistochemical staining, reported the overexpression of *MDM2* and *CDK4* in the oral cavity.^[15]^ Moreover, *MDM2* amplification has been reported in several cases of extra-pulmonary DDLS with IMT-like features.^[16,17]^ Based on these findings, *MDM2* fluorescence in situ hybridization (FISH) assay has been proposed as a diagnostic tool to differentiate undifferentiated pleomorphic sarcoma from various fibroinflammatory disorders.^[18,19]^ Since in our case, the microscopic presentation of IMT did not match WDLS or DDLS, the tumor may have arisen from stroma cells rather than adipose tissue. As 6 germline mutations were identified in our study, whether the genomic background predisposes a tumor towards malignant transformation remains to be elucidated.

In addition to *MDM2* and *CDK4*, we also identified gene amplification in *DDR2* in recurring IMT. DDR2 is a receptor tyrosine kinase that is activated by fibrillar collagens and might promote metabolism in adipocyte cells.^[20]^ Gene amplification and overexpression of *DDR2* has been reported in liposarcoma,^[21,22]^ and a variety of solid tumors.^[23,24]^ To our knowledge, ours is the first report to show *DDR2* overexpression in an IMT.

Although rare, radiotherapy has been reported to induce inflammatory changes and lead to IMT or sarcoma.^[25,26]^ The incidence of post-irradiation IMT or sarcoma after head-and-neck radiation therapy has been reported to be 0.02% and 0.20%, respectively.^[27,28]^ Post-radiation sarcoma (PRS) is often aggressive with poor prognosis.^[29]^ From a recent study,^[30]^ most cases of PRS (42 out of 43) were found to be high-grade tumors; the most common histology was osteosarcoma, followed by undifferentiated pleomorphic sarcoma and fibrosarcoma. None of the case series reported PRSs to be a liposarcoma. Although our case genetically mimicked liposarcoma and had no recurrence for >40 months following the second surgery, unlike PRS, we are unable to rule out the carcinogenic effects of radiotherapy on the genetic evolution in the tumor. It remains to be elucidated whether radiation therapy had any role to play in the newly developed *SDHC* gene amplification and *TSHR* p.Q720H mutation in recurring tumors.

## 4 Conclusions

IMT is currently classified as an intermediate and rarely metastasizing neoplasm. The diagnosis of *ALK*-negative IMT is challenging. Using massively parallel sequencing, we found that the majority of genetic changes in our case were gain in copy numbers with very few mutations. Although the case showed *MDM2*, *CDK4*, and *DDR2* gene amplification, which were genetically similar to liposarcoma, upon microscopic examination, neither the primary nor the recurrent IMTs presented as liposarcoma. Our case addressed the complexity of the histology presentation of IMT and highlights the importance of pathology/genomic correlation to define malignancy potential and clonal evolution of these tumors.

## Author contributions

**Conceptualization:** Tien-Chi Hou, Ying-Wen Su.

**Data curation:** Tien-Chi Hou, Pao-Shu Wu, Yu-Jen Chen, Shu-Jen Chen, Ying-Wen Su.

**Formal analysis:** Wen-Yu Huang, Shu-Jen Chen, Ying-Wen Su.

**Funding acquisition:** Yu-Jen Chen, Shu-Jen Chen, Ying-Wen Su.

**Investigation:** Tien-Chi Hou, Pao-Shu Wu, Wen-Yu Huang, Yi-Ting Yang, Kien Thiam Tan, Shih-Hua Liu, Shu-Jen Chen, Ying-Wen Su.

**Methodology:** Pao-Shu Wu, Yi-Ting Yang, Kien Thiam Tan, Ying-Wen Su.

**Project administration:** Yu-Jen Chen, Ying-Wen Su.

**Resources:** Pao-Shu Wu, Kien Thiam Tan, Shih-Hua Liu, Shu-Jen Chen, Ying-Wen Su.

**Software:** Yi-Ting Yang, Shu-Jen Chen.

**Supervision:** Yu-Jen Chen, Ying-Wen Su.

**Validation:** Tien-Chi Hou, Shih-Hua Liu.

**Writing – original draft:** Tien-Chi Hou, Ying-Wen Su.

**Writing – review & editing:** Tien-Chi Hou, Pao-Shu Wu, Wen-Yu Huang, Yi-Ting Yang, Kien Thiam Tan, Shih-Hua Liu, Yu-Jen Chen, Shu-Jen Chen, Ying-Wen Su.

Tien-Chi Hou orcid: 0000-0003-1090-038X.

Pao-Shu Wu orcid: 0000-0003-4823-9582.

Wen-Yu Huang orcid: 0000-0001-7129-8420.

Kien Thiam Tan orcid: 0000-0001-8854-0922.

Shih-Hua Liu orcid: 0000-0002-6928-9960.

Yu-Jen Chen orcid: 0000-0001-9794-8938.

Shu-Jen Chen orcid: 0000-0002-9666-8544.

Ying-Wen Su orcid: 0000-0003-3958-4202.
